# A case of digoxin intoxication caused by short-term massive overdose: Case report

**DOI:** 10.1097/MD.0000000000037034

**Published:** 2024-01-26

**Authors:** Hua Li, Chun-hai Zhang, Ming-wei Liu

**Affiliations:** a Department of Emergency, The Third People’s Hospital of Yunnan Province, Kunming, Yunnan, China; b Department of Emergency, The First Affiliated Hospital of Kunming Medical University, Kunming, Yunnan, China.

**Keywords:** arrhythmia, case reports, depression, digoxin intoxication, treatment

## Abstract

**Rationale:**

Digoxin is a frequently prescribed medication for the management of both acute and chronic cardiac insufficiency. The overdose ingestion of digoxin can result in a range of arrhythmias, with severe cases potentially leading to malignant arrhythmias and fatal outcomes. To date, there is a lack of documented cases related to acute digoxin intoxication resulting from the administration of massive digoxin overdose in the short term.

**Patient concerns:**

A 37-year-old female patient was admitted to the emergency department following a suicide attempt involving the administration of 330 tablets of digoxin (each tablet containing 0.25 mg). The patient exhibited symptoms of confusion, nausea, and vomiting for around 30 minutes. The patient had a history of depression.

**Diagnoses:**

The patient was diagnosed with digoxin intoxication.

**Interventions:**

The patient underwent many medical interventions including stomach lavage, administration of laxatives, correction of cardiac arrhythmias, provision of myocardial nutrition, diuresis, correction of acid-base balance, and management of electrolyte disturbances, among others.

**Outcomes:**

Following a treatment of 9 days, the patient exhibited no signs of discomfort, maintained consciousness, and the serum concentration of digoxin was indeterminable. Upon reevaluation of the electrocardiogram, it was determined that no arrhythmia was present. Consequently, the patient was authorized to be discharged from the hospital.

**Conclusions:**

There is currently no documented evidence of cases involving a significant overdose of digoxin resulting in intoxication. The patient had a comprehensive treatment regimen consisting of stomach lavage, administration of a laxative, correction of cardiac arrhythmias, provision of myocardial nutrition, fluid replacement, diuresis, and supportive therapy, resulting in successful outcomes.

**Lessons:**

There have been no known cases of intoxication resulting from a significant overdose of digoxin, specifically with the consumption of 330 tablets (0.25 mg/tablet). However, in the event of ingesting excessive amounts of digoxin, it is imperative to promptly administer stomach lavage, administration of a laxative, and arrhythmia correction. The administration of temporary pacemaker therapy is recommended for patients presenting with high atrioventricular block, whereas hemoperfusion is advised for patients with renal insufficiency as a means to eliminate digoxin from the body.

## 1. Introduction

Digoxin, a well-established pharmacological drug with cardiotonic properties, has a historical origin in the 18th century when it was initially employed by William Withering for the management of patients presenting with edema. Currently, digoxin is widely employed as a drug in the clinical management of chronic heart failure. Digoxin primarily acts as an inhibitor of the Na^+^-K^+^-ATPase enzyme located on the myocardial cell membrane. This mechanism of action leads to an augmentation of myocardial contractility, resulting in an elevation of the left ventricular ejection fraction in patients suffering from heart failure. Additionally, it contributes to a reduction in pulmonary capillary wedge pressure.^[1]^ Digoxin, on the one hand, has the ability to enhance vagal tone through the inhibition of Na^+^-K^+^-ATPase in the afferent nerve fibers of the vagus nerve. This action leads to a reduction in the automaticity of the sinoatrial node, a decrease in the conduction velocity of the atrioventricular node, and an extension of the effective refractory period. Consequently, the ventricular rate is slowed down in cases of atrial flutter and atrial fibrillation.^[2,3]^ On the other hand, it has the potential to reduce the duration of the atrial refractory period, accelerate the atrial rate, and enhance occult conduction, hence resulting in a deceleration of the ventricular rate.^[4]^ Therefore, digoxin is used in the treatment of heart failure through its positive inotropic effect and reducing the activity of the neuroendocrine system. However, it is worth noting that the therapeutic range of digoxin is characterized by a limited margin, as the therapeutic dosage closely approaches the toxic dosage. Furthermore, numerous factors exert an influence on this range, hence contributing to the occurrence of digoxin intoxication in clinics on an intermittent basis.

Digoxin intoxication frequently occurs during the treatment of chronic heart failure. The toxic threshold for digoxin concentration is commonly observed to range between 1.5 and 2.0 ng/mL. Although acute digoxin intoxication cases have been reported, acute digoxin intoxication resulting from an extremely high dose of digoxin (e.g., 330 tablets, each containing 0.25 mg) have not been reported in the literature. In this study, we report the diagnosis and treatment of a massive digoxin overdose intoxication case (330 tablets, each containing 0.25 mg), which will provide valuable guidance to doctors in effectively managing patients presenting with similar intoxication incidents.

## 2. Case report

### 2.1. Ethics approval and consent to participate

Informed written consent was obtained from the patient for the publication of this case report and the accompanying images. This study was reviewed and approved by the local ethics committee of First Affiliated Hospital of Kunming Medical University. Procedures followed were in accordance with the Helsinki Declaration of 1975, as revised in 2000.

### 2.2. Medical history

A 34-year-old female patient who was presented to the Emergency Department of the First Affiliated Hospital of Kunming Medical University by the 120 ambulance service for medical intervention following a suicide attempt involving the ingestion of 330 tablets of digoxin, each containing digoxin 0.25 mg. The motivation behind this act was attributed to her state of depression. She presented symptoms such as nausea, vomiting, and a period of unconsciousness lasting approximately 40 minutes.

### 2.3. Past medical history

The patient has a documented record of experiencing moderate to severe depression for 3 years and has been taking antidepressants irregularly. The patient negates any prior medical records indicating a history of diabetes, cardiovascular, and cerebrovascular diseases, lung and endocrine system disorders, as well as any significant organ diseases and infectious diseases. The patient exhibits a lack of reported injuries, surgical procedures, blood transfusions, allergies, and vaccination history.

### 2.4. Physical examination

The patient’s vital signs exhibited a body temperature of 36.6°C, a pulse of 100 beats per minute, a respiratory rate of 19 breaths per minute, and a blood pressure of 149/99 mm Hg. The overall state of health was suboptimal, and there was no observable enlargement of superficial lymph nodes throughout the entirety of the body. The craniofacial region had typical characteristics, with a Glasgow Coma Index Score of 12. The pupils were symmetrical and exhibited a diameter of 3 mm, accompanied by the presence of both direct and indirect light responses. However, the light reflexes were seen to be diminished in intensity. Both eyes could move in all directions, and no enlargement of the thyroid gland was detected. During chest auscultation, it was seen that there was thoracic deformity and coarse respiratory sound in both lungs with the absence of dry and moist rales. Cardiac examination was unremarkable with no observed enlargement of the heart border. The heart rate was measured at 100 beats/min, exhibiting regular rhythm, and no pathological murmurs were auscultated in the valves. The abdominal region had a flat and soft appearance, without any tenderness or rebound pain. The liver was not palpable, and the bowel sounds were within the expected range.

### 2.5. Laboratory data

The laboratory tests were performed on September 4, 2023. The results of the analysis revealed the following values: white blood cell count (WBC), 5.92 × 10^9^/L; neutrophils, 78.3%; lymphocytes, 9.9%; red blood cell count, 5.64 × 10^12^/L; hemoglobin, 159 g/L; platelet count, 349 × 10^9^/L; plateletcrit, <0.05 mg/L; and C-reactive protein, 6.7 pg/L. The levels of myocardial enzymes observed in the patient’s blood sample were as follows: lactate dehydrogenase, 237 U/L; creatine phosphate kinase, 184.2 U/L; myoglobin, 106 ng/mL; α-eOB-hydroxybutyrate dehydrogenase, 244.5 U/L; creatine phosphate kinase-isenzymes, 4.2 U/L; troponin, 0.119 ng/mL; and B-type natriuretic peptide, 37 pg/mL. The renal function parameters were measured as follows: blood urea nitrogen, 11.0 mmol/L; creatinine, 84 μmol/L. The electrolyte levels in the serum were as follows: chloride, 93.56 mmol/L; calcium, 2.02 mmol/L; sodium, 138.48 mmol/L; and potassium, 3.74 mmol/L. The results of the liver function tests revealed that the alanine aminotransferase, 34.8 U/L; the aspartate aminotransferase, 28.2 U/L; total protein, 57.8 g/L, albumin, 34.67 g/L. The blood gas analysis revealed the following results: the pH 7.42; the partial pressure of carbon dioxide, 36.8 mm Hg; partial pressure of oxygen, 80 mm Hg; actual bicarbonate, 32.2 mmol/L; base excess, 6.4 mmol/L; sodium ion concentration (Na^+^), 137.7 mmol/L; chloride ion concentration (Cl^−^), 92 mmol/L; blood lactate, 1.35 mmol/L. The serum levels of digoxin exceeded 7 ng/mL. The electrocardiogram revealed a heart rate of 100 beats per minute and a pattern consistent with atrial flutter. The coagulation and fibrinolysis system was assessed, revealing a fibrin degradation product, 1.4 mg/L; D-dimer, 0.49 mg/L; antithrombin III, 85%; prothrombin time, 11.7 seconds; prothrombin activity, 126%; international standard ratio, 0.89; prothrombin time ratio, 0.91; reagent sensitivity index, 1.29; fibrinogen, 4.29 g/L; clotting time, 19.7 seconds; activated partial thromboplastin time, 45.1 second.

### 2.6. Diagnosis and treatment

**Figure 1.**
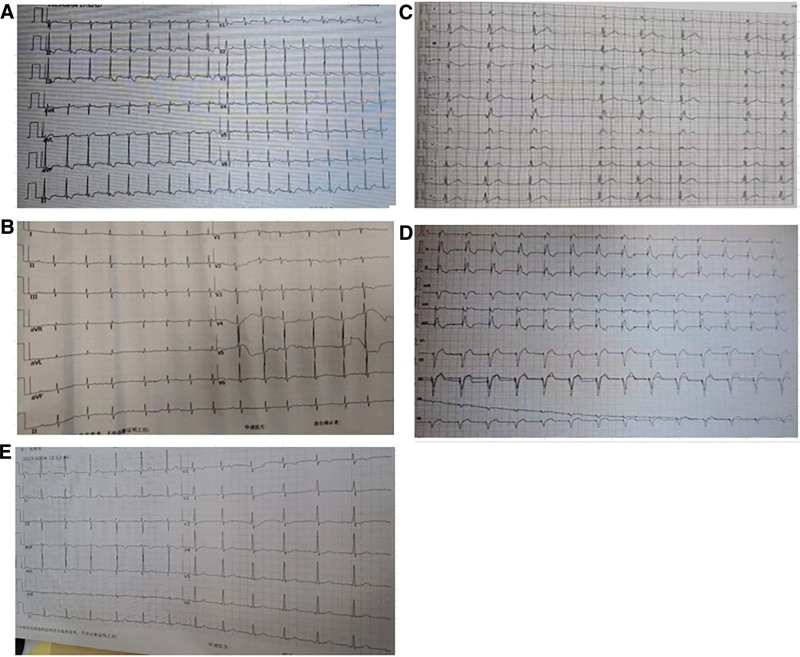
ECG changes in patient during treatment. (A) ECG at admission; (B) ECG on the second day after admission; (C) ECG on the third day after admission; (D) ECG after implantation of a temporary pacemaker on the third day after admission. (E) ECG after withdrawal of the temporary pacemaker on the seventh day of admission. ECG = electrocardiogram.

The patient was diagnosed with digoxin intoxication based on patient’s medical history, presenting symptoms and signs, as well as the measured serum concentration of digoxin. The patient had immediate medical interventions including stomach lavage, administration of a laxative, correction of cardiac arrhythmias, provision of myocardial nutritional support, fluid replacement and diuresis, correction of acid-base balance, and management of electrolyte disturbances, among other treatments. At the same time, dynamic monitoring techniques were applied to assess both the plasma concentration of digoxin and any associated electrocardiographic changes. The concentration of digoxin exhibited a progressive reduction over the duration of the treatment, ultimately returning to a normal level after 1 week of treatment, as indicated in Table 1. However, the electrocardiography results indicated the presence of atrial flutter on admission (Fig. 1A), atrial fibrillation rhythm on the second day (Fig. 1B), and grade II, type I atrioventricular block on the third day (Fig. 1C). In response, the patient received immediate therapy in the form of a temporary pacemaker implantation, which was confirmed by the pacing rhythm observed on electrocardiography (Fig. 1D). On the seventh day following admission, once the concentration of digoxin had returned to a normal level, the temporary pacemaker was removed and the electrocardiogram displayed a normal reading (Fig. 1E). The patient was authorized to be discharged after 7 days of hospitalization.

**Table 1:** Changes in the blood concentration of digoxin in patient during treatment.

Date of measurement	September 5, 2023	September 6, 2023	September 7, 2023	September 8, 2023	September 9, 2023	September 11, 2023
Digoxin (ng/mL)	>7	2.43	1.21	0.97	0.42	0.27

### 2.7. Follow-up after discharge

One-week after the treatment, the patient reported the absence of any discomfort and was recommended to maintain regular administration of the medication.

## 3. Discussion

Digoxin is a cost-effective, efficacious, and widely used drug for the treatment of heart failure, especially in patients with systolic heart failure, atrial fibrillation, and rapid ventricular rates. Following oral administration, the drug undergoes absorption in the upper small intestine with an absorption rate of 75%. The onset time ranges from 0.5 to 2.0 hours, while the maximum effect time ranges from 2 to 6 hours. The drug exhibits low plasma protein binding rate at 20% to 25%. The apparent volume of distribution is between 6 and 10 L/kg, and the half-life of the drug is 36 hours.^[5,6]^

The diagnostic indicators of digoxin intoxication can be categorized into following key aspects^[7]^: initial gastrointestinal manifestations and accompanying systemic symptoms include but are not limited to nausea, vomiting, diarrhea, anorexia, dizziness, fatigue, and insomnia. Cardiotoxicity refers to the occurrence of novel arrhythmias that cannot be attributed to the underlying pathology. There are other visual alterations that patients may experience, including yellow vision, green vision, and blurred vision. Nervous system signs, including but not limited to headache, disorientation, delirium, and dizziness, are observed. The serum concentration of digoxin exceeds 2.0 μg/L. In this case, the patient exhibited symptoms and signs, including nausea, vomiting, fatigue, and lethargy, that were indicative of digoxin overdose. Given that a majority of patients utilizing cardiac digitalis preparations exhibit organic heart disease, it is common for them to experience arrhythmias, including newly developed ones, while undergoing treatment with cardiac digitalis. Following discontinuation of medication or the implementation of symptomatic interventions, there is typically an observed improvement.^[7]^ However, in this patient, the administration of a large dose of digoxin orally at one time resulted in the toxic reaction of arrhythmias and the manifestation of neurological impairment such as sleepiness. Efforts were made to facilitate the elimination of digitalis from the body and to manage the occurrence of arrhythmias.

Once digoxin intoxication occurs clinically, the initial course of treatment typically involves discontinuing or reducing the dosage of the digoxin. Additionally, prompt administration of stomach lavage and induction of vomiting is recommended in instances of high dosage intake. The second approach involves the elimination of influential factors, enhancement of myocardial ischemia and hypoxia, correction of electrolyte abnormalities, and prevention of drug interactions.^[8]^ In cases when the patient’s symptoms and signs fail to show improvement or the blood concentration of digoxin remains elevated, it is imperative to immediately initiate hemoperfusion.^[9]^ Digoxin is a pharmacological compound characterized by its lipophilic nature, which contributes to its prolonged half-life and delayed elimination by renal excretion in cases of intoxication. Hemoperfusion is primarily employed for the purpose of eliminating toxins from the body by adsorbent adsorption facilitated by extracorporeal circulation, hence mitigating toxicity. One of its notable advantages is in its wide-ranging scavenging capabilities, which encompass a diverse array of toxins. Furthermore, it has a particularly effective scavenging ability toward lipophilic toxins, as well as scavenging effect on free poisons in the blood.^[10]^ According to the findings of the study, subsequent to the process of adsorption, digoxin exhibits a broad distribution throughout the body, with a myocardial tissue concentration that is approximately 30 times higher than that observed in the blood. Hemoperfusion has the potential to decrease the blood concentration of digoxin, leading to the release of digoxin from tissues into the bloodstream, hence causing a subsequent increase in serum concentration of digoxin. Hence, it is imperative to administer numerous sessions of hemoperfusion to patients experiencing digoxin intoxication, as this therapeutic intervention facilitates a gradual reduction in the serum concentration of digoxin.^[11]^ Achenbach et al^12^ reported massive digoxin overdose intoxication, especially in patients with renal failure. The patients received immediate hemoperfusion treatment, resulting in favorable outcomes. The patients shown improvement and were subsequently discharged from the hospital.^[12,13]^ However, most of these studies did not measure the serum concentration of digoxin before and after hemoperfusion, thereby necessitating further inquiry into the impact of hemoperfusion on the therapeutic effectiveness of digoxin in severe cases. In this study, attributed to the patients’ youthful age and normal renal function, extensive fluid administration and diuresis to promote digoxin elimination also had favorable outcomes. High-dose digoxin intoxication has been found to frequently result in arrhythmias, particularly ventricular conduction block, but arrhythmias associated with heightened autonomic activity are rather uncommon.^[14,15]^ The utilization of temporary cardiac pacemaker implantation has demonstrated efficacy in the management of diverse arrhythmias resulting from digoxin toxicity. Therefore, the patient here who experienced the onset of type II atrioventricular block over the course of treatment was treated with temporary pacemaker implantation for remedial purposes.

### 3.1. Strengths and limitations

#### 3.1.1. Strengths.

There is currently no documented evidence of cases involving a significant overdose of digoxin resulting in intoxication. The patient had a comprehensive treatment regimen resulting in successful outcomes.

#### 3.1.2. Limitations.

Limitations in the treatment of this patient: since the patient’s youthful age and normal renal function, a large amount of fluid replacement and diuresis were given to promote the elimination of digitalis with good outcomes. However, there were deficiencies observed in the treatment procedure. Given the patient’s high oral digoxin dosage, it could have been advantageous to provide early hemoperfusion treatment as part of their therapeutic regimen. Regrettably, hemoperfusion treatment was not executed. The patient presented with a documented history of depression. Despite the administration of antidepressant therapy throughout the treatment period, there was no assessment of antidepressant drug levels.

## 4. Conclusions

In summary, there is currently no existing study available regarding the massive digoxin overdose intoxication (330 tablets, 0.25 mg/tablet). In the case of patients experiencing severe digoxin intoxication with super-high levels and normal renal function, the implementation of fluid replacement, diuretic therapy to expedite the elimination of digoxin, management of arrhythmias, and the utilization of a temporary cardiac pacemaker had demonstrated specific clinical outcomes. This study is limited to a small sample size and still requires the collection of additional samples in the future to provide controlled analysis and further research.

## Acknowledgments

We thank Home for Researchers editorial team (www.home-for-researchers.com) for language editing service.

## Author contributions

**Data curation:** Hua Li.

**Investigation:** Hua Li, Ming-wei Liu.

**Supervision:** Hua Li, Chun-hai Zhang.

**Writing – original draft:** Hua Li, Ming-wei Liu.

**Conceptualization:** Chun-hai Zhang, Ming-wei Liu.

**Formal analysis:** Chun-hai Zhang.

**Methodology:** Chun-hai Zhang.

**Resources:** Chun-hai Zhang.

**Funding acquisition:** Ming-wei Liu.

**Project administration:** Ming-wei Liu.

**Software:** Ming-wei Liu.

**Visualization:** Ming-wei Liu.

**Writing – review & editing:** Ming-wei Liu.
